# Novel insights into gut microbiota alterations in major depressive disorder with suicidal ideation: a metagenomic analysis

**DOI:** 10.3389/fmicb.2026.1843301

**Published:** 2026-06-10

**Authors:** Lulu He, Yuanyuan Huang, Hehua Li, Baoyuan Zhu, Ziyun Zhang, Jingping Wu, Sumiao Zhou, Qianqian Zhan, Kai Wu, Fengchun Wu

**Affiliations:** 1 The Affiliated Brain Hospital, Guangzhou Medical University, Guangzhou, China; 2 Guangdong Engineering Technology Research Center for Translational Medicine of Mental Disorders, Guangzhou, China; 3 School of Biomedical Sciences and Engineering, Guangzhou International Campus, South China University of Technology, Guangzhou, China

**Keywords:** *Bacteroides cellulosilyticus*, gut microbiome, major depressive disorder, metagenome, suicidal ideation

## Abstract

**Introduction:**

Suicidal ideation in major depressive disorder (MDD) is common, yet its biological mechanisms and biomarkers remain unclear. The gut microbiota, a key component of the gut-brain axis, has been implicated, but current evidence is limited.

**Methods:**

We analyzed fecal samples from 141 participants, including 52 healthy controls (HCs) and 89 first-episode, drug-naïve MDD patients, further classified into suicidal ideation (SI, *n* = 57) and non-suicidal ideation (NSI, *n* = 32) groups using the Beck Scale for Suicide Ideation (BSSI). Shotgun metagenomic sequencing with HUMAnN3-based taxonomic and functional profiling was performed. Microbial diversity, differential abundance, and partial correlation analyses with suicidal ideation severity were conducted to identify key microbial taxa associated with suicidal ideation. For functional difference analysis, MaAsLin2 was employed across four levels: KEGG Orthology (KO), KEGG pathways, CAZy, and MetaCyc pathways. Mediation analysis was used to assess potential mediating effects between suicidal ideation and key microbial taxa after adjustment for age, sex, education, and BMI.

**Results:**

No significant differences were observed in overall microbial diversity. *Bacteroides cellulosilyticus* was enriched in HCs and showed a significant negative association with suicidal ideation severity. Functionally, compared with the NSI group, patients with suicidal ideation exhibited reduced microbial capacities related to peptidoglycan biosynthesis. Mediation analysis further indicated that *B. cellulosilyticus* may modulate suicidal ideation through pathways involved in carbohydrate transport and metabolism, vitamin K2 biosynthesis, and DNA repair.

**Conclusion:**

*Bacteroides cellulosilyticus* may act as a potentially protective microbial species, negatively regulating suicidal ideation, possibly by enhancing carbohydrate metabolism and short-chain fatty acid production. Notably, this species has received limited attention in the context of psychiatric disorders, highlighting its potential as a novel microbial target. These findings provide new microbiome-based insights into suicidal ideation in MDD.

## 1 Introduction

Major depressive disorder (MDD) is one of the most common psychiatric disorders, characterized by high rates of suicide and disability, and represents a major component of the global disease burden (Huang et al., 2019; He L. et al., 2025). The World Health Organization has projected that by 2030, depression may surpass heart disease to become the leading cause of disease burden worldwide (World Health Organization [WHO], 2026). The most severe consequence of MDD is suicide, with a lifetime prevalence of suicidal ideation ranging from 18.0 to 58.0% among patients (Cai et al., 2021; Dong et al., 2018). Suicide has become a major global public health concern and was the fourth leading cause of death among individuals aged 15–29 years in 2019 (Kara and Gülbahçe, 2024). Notably, most individuals experiencing suicidal ideation do not seek or receive appropriate help (Milton et al., 2020; O’Connor and Nock, 2014), underscoring the importance of identifying early biomarkers and developing comprehensive intervention strategies.

The occurrence of suicidal behavior involves complex interactions among multiple factors, and the ability to predict the transition from suicidal ideation to suicide attempts remains limited (Carter and Spittal, 2018). The stress–diathesis model suggests that suicidal behavior arises from the interaction between environmental stressors and individual vulnerability (Hawton and van Heeringen, 2009; Zhang et al., 2026). Previous studies have implicated several biological factors in suicide, including systemic inflammation (Lindqvist et al., 2009; Wu J. et al., 2026; Wu F. et al., 2026; Zhu et al., 2026; Chen S. et al., 2026), neuroinflammation (Dickerson et al., 2017; García-Gutiérrez et al., 2025; Neupane et al., 2023; Sha et al., 2024; Sudol and Mann, 2017), specific polymorphisms in the brain-derived neurotrophic factor (BDNF) gene (Kang et al., 2020), and sleep disturbances (Geoffroy et al., 2021). However, single factors have limited explanatory power, highlighting the need to integrate multidimensional biomarkers to improve predictive accuracy (Thompson et al., 2021; Chen B. et al., 2026). In recent years, the gut microbiota, as a key component of the gut–brain axis, has been increasingly linked to psychiatric disorders and suicidal ideation, and may serve as a complementary indicator for suicide risk assessment (Huang et al., 2024; Li et al., 2025; Morena et al., 2025; Ohlsson et al., 2019).

Emerging evidence has revealed associations between gut and oral microbiota and suicide-related phenotypes. For example, increased abundance of *Veillonella* in saliva has been observed in young individuals with suicidal ideation, and specific genetic loci have been associated with microbial alterations (Ahrens et al., 2022). In adolescents, *Mitsuokella* has been reported to distinguish individuals with non-suicidal self-injury from HCs (Cai et al., 2022). Additionally, several studies have identified microbial alterations in patients with depression or suicidal behaviors, including reduced abundance of *Dorea* and *Faecalibacterium* (Maes et al., 2024), and positive associations between *Phascolarctobacterium* and the severity of suicidal ideation (Chen and Wu, 2025). Other taxa, such as members of Erysipelotrichaceae and the *Eubacterium hallii group*, have also been linked to suicidal ideation (Xu et al., 2025). At the functional level, individuals completed suicide exhibit enrichment of pathways related to inflammation, infection, and antibiotic resistance (Li et al., 2026), whereas alive MDD show enrichment in energy metabolism and vitamin biosynthesis pathways (Kozhakhmetov et al., 2025). Metabolic differences primarily involve energy, lipid, and amino acid metabolism (Prandovszky et al., 2025). However, some studies have not found significant associations between gut microbiota and suicidal ideation (Thompson et al., 2021).

Despite these findings, several limitations remain. Many studies have small sample sizes and limited statistical power. Most rely on 16S rRNA sequencing, which offers limited taxonomic resolution and infers function indirectly, potentially reducing accuracy. In addition, the inclusion of heterogeneous psychiatric conditions, such as both depression and bipolar disorder, may obscure true associations (Thompson et al., 2021). In contrast, metagenomic sequencing enables species- or even strain-level identification and direct functional profiling, providing a more powerful approach to elucidate the role of the gut microbiota in suicidal ideation.

Based on this background, the present study employed metagenomic sequencing to systematically characterize the composition and functional features of the gut microbiota in MDD patients with and without suicidal ideation, while controlling for demographic variables. By integrating taxonomic and functional data, we aimed to identify key microbial species and potential biological mechanisms associated with suicidal ideation, thereby providing novel biomarkers for early risk identification and a theoretical basis for gut–brain axis–based interventions.

## 2 Materials and methods

### 2.1 Study design and participants

This study was approved by the Ethics Committee of the Affiliated Brain Hospital of Guangzhou Medical University, and all participants provided written informed consent prior to enrollment. Patients were recruited from outpatient and inpatient departments, while HCs were recruited through public advertisements and online platforms. All participants were Han Chinese from Guangdong Province, with relatively similar dietary habits.

Inclusion criteria were as follows: (1) meeting the diagnostic criteria for MDD according to the Diagnostic and Statistical Manual of Mental Disorders, Fifth Edition (DSM-5); (2) age between 18 and 45 years; (3) Han ethnicity; (4) first-episode patients with illness duration < 2 years; (5) no history of psychotropic medication use; (6) and a 17-item Hamilton Depression Rating Scale (HAMD-17) score ≥ 17.

Exclusion criteria included: presence of organic brain disorders or severe physical illnesses; history of head trauma or loss of consciousness; comorbid conditions affecting emotional state (e.g., substance abuse, thyroid dysfunction, anemia); pregnancy or lactation; gastrointestinal diseases (including diarrhea) or antibiotic use within the past 3 months.

Sample size estimation: Sample size calculation indicated that, at a significance level of 0.05 and 80% statistical power, 159 participants (53 per group) were required to detect a medium effect size (Cohen’s *f* = 0.25). A total of 141 participants were ultimately included (HC: 52, SI: 57, NSI: 32). The relatively smaller sample size in the NSI group was mainly due to exclusion during fecal sample quality control (e.g., low sequencing quality or insufficient reads after host DNA removal).

### 2.2 Clinical assessments

Demographic data [sex, age, years of education, and body mass index (BMI)] and clinical information were collected.

The primary clinical endpoint of this study was suicidal ideation, assessed using the Beck Scale for Suicide Ideation (BSSI). Suicidal ideation over the past week was assessed using the BSSI. The BSSI consists of 19 items, each scored from 0 to 2, with a total score ranging from 0 to 38. Participants were classified into the suicidal ideation group (SI, *n* = 57) if they scored > 0 on item 4 (active suicidal desire) or item 5 (passive suicidal desire); otherwise, they were assigned to the non-suicidal ideation group (NSI, *n* = 32). The total BSSI score was used as a continuous measure to quantify the severity of suicidal ideation, with higher scores indicating greater severity (He Y. et al., 2025; Wang et al., 2025).

Depressive symptom severity was evaluated using the HAMD-17 and was used for eligibility assessment. All assessments were conducted by trained psychiatrists who had undergone standardized training.

Regarding missing data, participants with missing key clinical variables (e.g., BSSI or HAMD-17 scores) were excluded from the final analysis. For variables with minimal missingness (e.g., BMI), missing values were imputed using the mean of the observed values.

### 2.3 Metagenomic sequencing data processing and quality control

Fecal samples were collected from participants’ first bowel movement in the morning under fasting conditions. To ensure sample integrity and prevent contamination, all collections were performed by trained personnel using sterile tools. Samples were transported within 1 h and stored at –80°C until metagenomic analysis. Detailed procedures for DNA extraction, library construction, and sequencing are provided in Supplementary material 1.

Raw metagenomic sequencing data (FASTQ format) were first assessed using FastQC (version 0.12.1). Adapter trimming and quality filtering were performed using fastp (version 0.21.0). Subsequently, KneadData (version 0.12.0) was used for comprehensive quality control and host DNA removal. Within the KneadData pipeline, Trimmomatic (version 0.39) was applied to remove low-quality reads (sliding window: 4 bp; average Phred score < 20) and short reads ( < 50 bp). Remaining reads were aligned to the human reference genome (hg37dec_v0.1) using Bowtie2 (version 2.5.1) with the parameters “–very-sensitive” and “–dovetail” to remove host-derived sequences.

### 2.4 Taxonomic and functional annotation

High-quality non-host reads were concatenated and analyzed using HUMAnN3 (version 3.7) for taxonomic and functional profiling. Taxonomic composition was determined using MetaPhlAn4 (version 4.0.6) integrated within HUMAnN3, based on clade-specific marker genes. Functional annotation was performed by aligning reads to the UniRef90 protein database using DIAMOND.

Gene family abundances (UniRef90) were regrouped into KEGG Orthology (KO) identifiers using the humann_regroup_table utility. To account for variations in sequencing depth, KO abundances were normalized to copies per million (CPM) using humann_renorm_table. For higher-level functional categorization, KO abundances were further aggregated into KEGG functional hierarchies (Levels 1–3) based on a predefined mapping database, facilitating a multi-tiered assessment of microbial metabolic potential. Additionally, microbial metabolic pathways were reconstructed and quantified based on the MetaCyc database using the core HUMAnN3 pipeline. To further characterize the specialized metabolic potential of the gut microbiota, gene families were also annotated against the Carbohydrate-Active EnZymes (CAZy) database. This comprehensive approach facilitated a multi-tiered assessment of microbial metabolic and enzymatic potential across the study groups.

### 2.5 Microbial diversity analysis

Alpha diversity indices, including Observed richness (to measure community complexity), Shannon index, and Simpson index (to evaluate both richness and evenness), were calculated using the R package vegan (version 2.6–4). These complementary metrics were selected to provide a comprehensive assessment of microbial diversity, ensuring that potential shifts in both rare and dominant taxa were captured. To account for potential confounding factors, differences in alpha diversity among the HC, NSI, and SI groups were assessed using linear models (LM) with Type III Sum of Squares, incorporating age, sex, BMI, and education level as covariates.

Beta diversity was evaluated based on Bray–Curtis distances, a metric chosen for its sensitivity to both taxonomic composition and relative abundance. The microbial community structure was visualized using Principal Coordinate Analysis (PCoA). To test the significance of group differences while controlling for demographic and clinical confounders (age, sex, BMI, and education), Permutational Multivariate Analysis of Variance (PERMANOVA) was performed using the adonis2 function with 999 permutations. All statistical analyses and visualizations were conducted in R (version 4.3.1) using the ggplot2 (version 3.4.2), ggpubr (version 0.6.3), and patchwork packages (version 1.3.2).

### 2.6 Identification of key microbial species associated with suicidal ideation

Differential taxa were identified using LEfSe (version 1.1.2, local implementation) (LDA > 2.0, *p* < 0.05). This approach utilizes a hierarchy of statistical tests: a Kruskal–Wallis test to identify features with significantly different abundances, followed by Wilcoxon rank-sum tests to assess biological significance. The differential analysis was conducted independently at the phylum, genus, and species levels. The final ranking of biomarkers was based on LDA scores, providing a robust measure of the effect size for each differentially abundant taxon or pathway. To validate the robustness of these findings and account for the compositional nature of metagenomic data, differential abundance analysis was performed at the species level using MaAsLin2 (version 1.15.1). For MaAsLin2, features with prevalence < 10% were excluded, and data were Centered Log-Ratio (CLR) transformed. Linear models were fitted by adjusting for age, sex, BMI, and education as fixed effects, with a significance threshold of *q* < 0.1.

To reduce sparsity and compositional bias, low-prevalence taxa (present in < 20% of samples) were excluded prior to analysis. The remaining species were then subjected to CLR transformation. Partial correlation analyses were performed to assess the associations between microbial abundance and clinical scores (BSSI total score). To control for potential confounding factors, sex, age, BMI, and years of education were included as covariates in the models. These analyses were implemented using the ppcor package (version 1.1) in R. The correlation coefficients (*r*) and nominal *p*-values were reported to characterize the directional trends between specific taxa and suicidality-related phenotypes.

### 2.7 Differential functional analysis

Differential functional analysis was conducted using the R package MaAsLin2 (version 1.15.1) (Mallick et al., 2021). To comprehensively characterize the functional landscape, we performed comparisons across four distinct levels: unstratified KEGG Orthology (KO) groups, KEGG functional hierarchies (Levels 1–3), MetaCyc pathways, and Carbohydrate-Active EnZymes (CAZy). Input abundances were preprocessed by removing features with < 10% prevalence, followed by CLR transformation.

Age, sex, BMI, and years of education were included as fixed-effect covariates. The analysis utilized the same linear model (LM) settings and parameters as described in section 2.6 (Identification of key microbial species associated with suicidal ideation). Multiple testing correction was performed using the Benjamini-Hochberg (BH) method, with a significance threshold of *q* < 0.1 applied to minimize false positives. All functional results were reported at both the KO and pathway levels, and data visualization was conducted in R using ggplot2 (version 3.4.2).

### 2.8 Mediation analysis

Mediation analysis was performed using the mediation package (version 4.5.1) in R. CLR-transformed key microbial taxa were treated as independent variables, while BSSI scores were considered as dependent variables. KOs contributed by the key microbial taxa were selected as mediators. Total, direct, and indirect effects were estimated using 1,000 bootstrap iterations. Given the exploratory nature of the study, no multiple testing correction was applied, and results should be interpreted with caution. The average causal mediation effect (ACME) and total effect were considered significant at *p* < 0.05.

### 2.9 Statistical analysis

Statistical analyses of demographic and clinical data were performed using SPSS (version 27.0, IBM Corp., Armonk, NY, United States). Categorical variables were compared using the chi-square test. The distribution of continuous variables was assessed for normality, and group differences were analyzed using the Student’s *t*-test, Mann–Whitney U test, or Kruskal–Wallis test, as appropriate. For multiple comparisons, *post-hoc* pairwise tests were conducted with Bonferroni correction to control for Type I error. A two-tailed *p* < 0.05 was considered statistically significant.

## 3 Results

### 3.1 Demographic and clinical characteristics of participants

No significant differences were observed among the three groups (HC, NSI, SI) in sex distribution (*p* = 0.205) or BMI (*p* = 0.913). However, significant differences were found in age (*p* = 0.009) and years of education (*p* = 0.003). *Post-hoc* pairwise comparisons with Bonferroni correction indicated that the NSI group was older than both the SI and HC groups, while the HC group had more years of education than the SI group (Table 1).

**TABLE 1:** Demographic and clinical characteristics.

Characteristics	HC (*n* = 52)	NSI (*n* = 32)	SI (*n* = 57)	Statistics	*P*	*Post-hoc* comparisons*^a^*
Sex (male/female)	26/26	14/18	10/38	χ^2^ = 3.166	0.205	/
Age	22 (20, 23)	25 (22, 28)	22 (20, 24.5)	*H* = 9.397	**0.009**	NSI>SI*,NSI>HC*
BMI	20.96 (18.86, 22.92)	20.13 (18.52, 24.42)	20.45 (18.45, 23.79)	*H* = 0.181	0.913	/
Years of education	16 (14, 17)	16 (12, 16)	15 (12, 16)	*H* = 11.812	**0.003**	HC>SI*
HAMD-17	/	21.63 ± 4.46	23.84 ± 4.22	*t* = -2.331	**0.022**	/
BSSI total	/	1 (0, 3)	15 (10.5, 21)	W = 1781.5	**<0.001**	/

*^a^* Bonferroni corrected. Bold values indicate statistically significant *p*-values; * indicates significance after multiple testing correction. Normally distributed data are presented as mean ± standard deviation (SD), whereas non-normally distributed data are expressed as median (interquartile range, IQR: 25th–75th percentiles). HAMD-17 and BSSI scores were only assessed in MDD patients (NSI and SI). χ^2^, Chi-square test; H, Kruskal–Wallis; *t*-test; W, Mann–Whitney U; HC, Healthy Control Group; SI, Major Depressive Disorder with Suicidal Ideation; NSI, Major Depressive Disorder without Suicidal Ideation.

Clinical assessments showed that the SI group had significantly higher HAMD scores (*p* = 0.022) and BSSI scores (*p* < 0.001) compared with the NSI group (Table 1).

### 3.2 Gut microbiota diversity analysis

**FIGURE 1:**
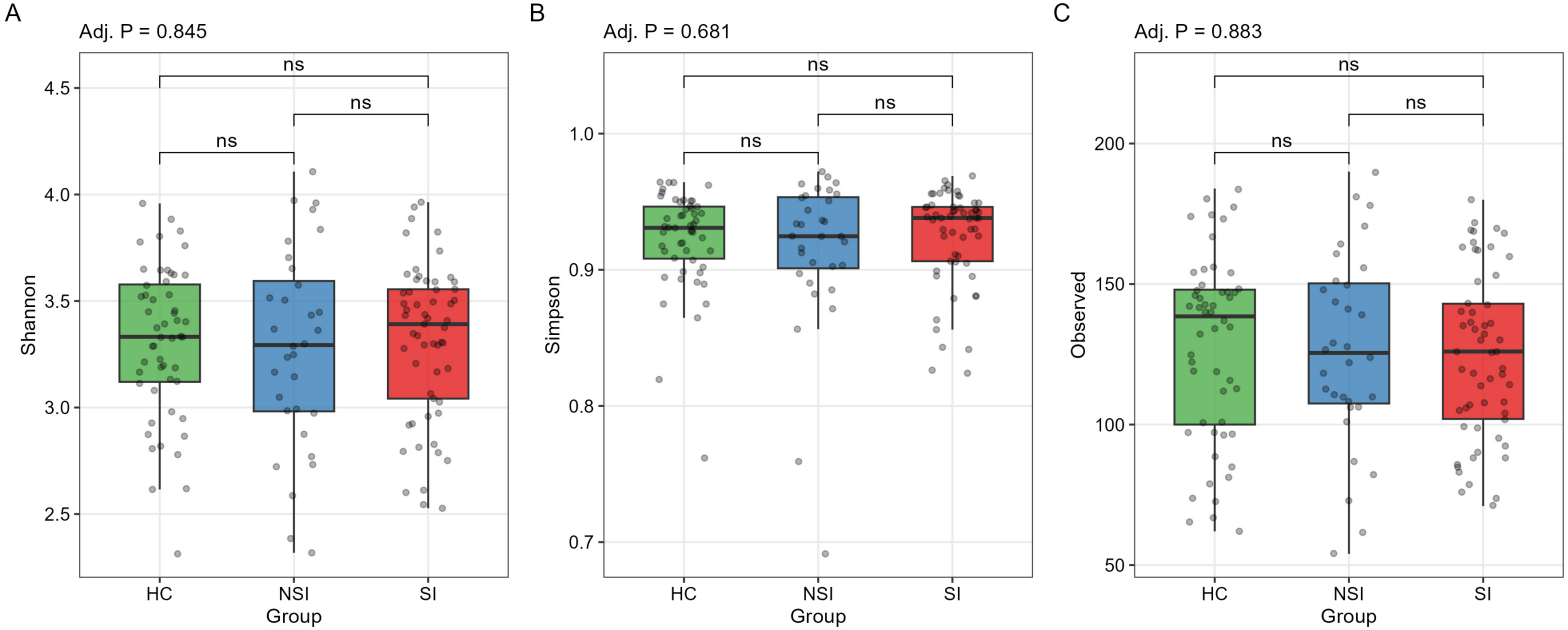
Microbial alpha diversity across groups. Alpha diversity indices, including **(A)** Shannon, **(B)** Simpson, and **(C)** Observed richness, were compared among the HC, NSI, and SI groups at the species level. Statistical significance was assessed using linear models (LM) adjusting for age, sex, BMI, and education level as covariates. Adjusted *p*-values (Adj. P) are shown above each plot, with “ns” indicating no significant differences between pairs *(p* > 0.05).

**FIGURE 2:**
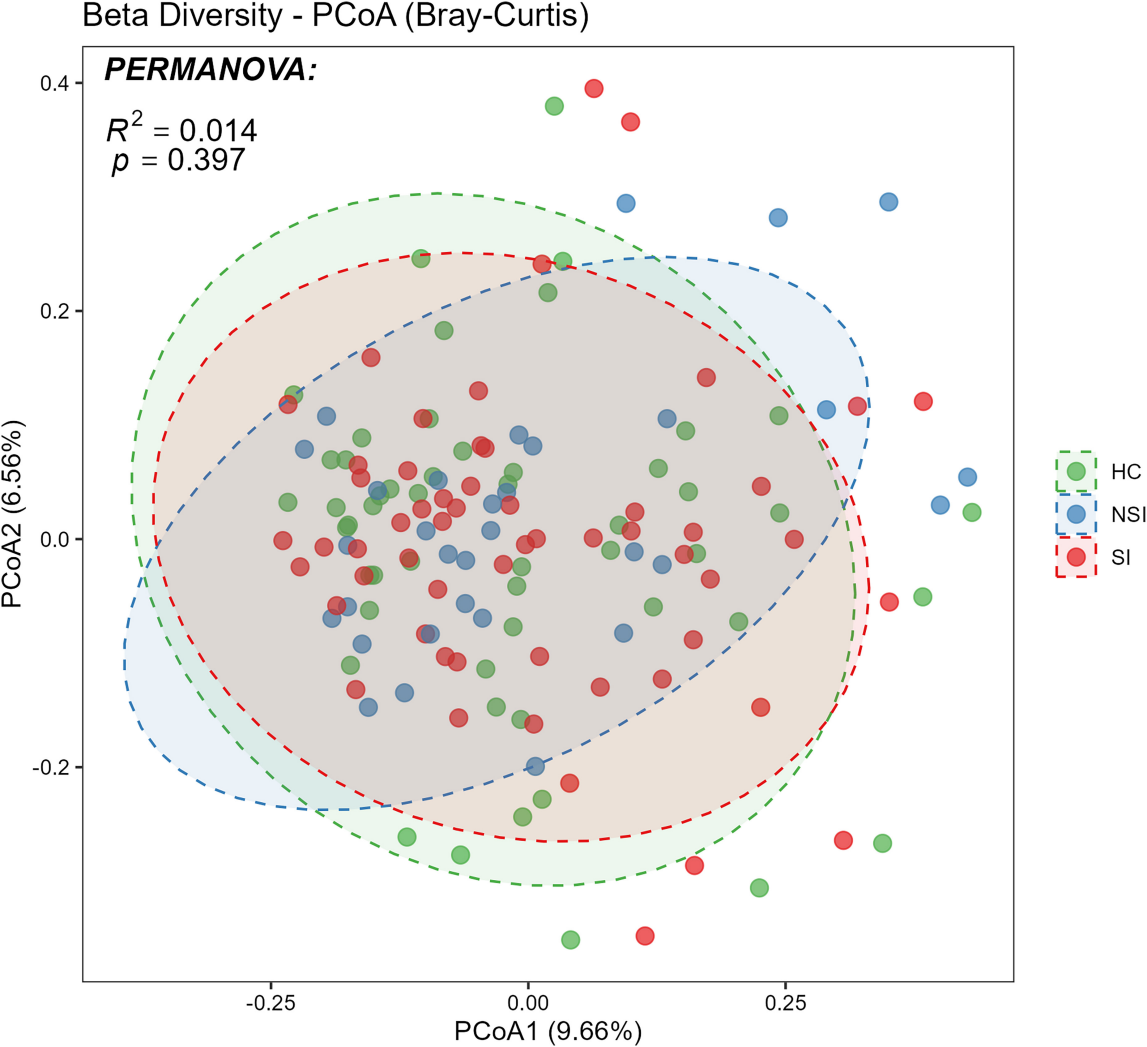
Microbial community structure across groups. Principal coordinate analysis (PCoA) based on Bray-Curtis distances illustrates the microbial community composition among the SI (red), NSI (blue), and HC (green) groups. PCoA1 and PCoA2 explain 9.66 and 6.56% of the total variance, respectively. PERMANOVA analysis (*R*^2^ = 0.014, *p* = 0.397), performed using the adonis2 function while adjusting for age, sex, BMI, and education level, indicates no significant separation in overall community structure among the three groups.

After adjusting for age, sex, BMI, and education level, no significant differences in alpha diversity at the species level were observed among the three groups (Figure 1; all adjusted *p* > 0.05, Supplementary Table 1). PCoA based on Bray–Curtis distance, with covariate adjustment also showed no significant separation of the microbial communities (Figure 2; *R*^2^ = 0.014, *p* = 0.397, Supplementary Table 2), suggesting that microbial alterations were primarily reflected at the level of specific taxa rather than the overall community structure.

### 3.3 Identification of the key microbial species: *Bacteroides cellulosilyticus*

**FIGURE 3:**
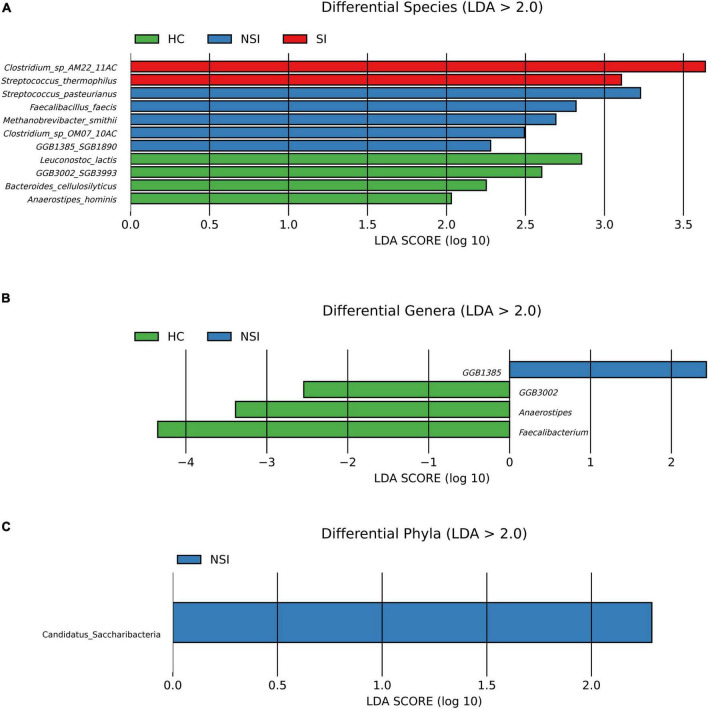
Microbial taxonomic distinctions identified by LEfSe analysis. Differentially abundant taxa among the SI (red), NSI (blue), and HC (green) groups were identified using LEfSe across multiple taxonomic ranks: **(A)** species level, **(B)** genus level, and **(C)** phylum level. Significant differences were determined using the Kruskal-Wallis test (*p* < 0.05), and only taxa with a Linear Discriminant Analysis (LDA) score (log_10_) greater than the threshold of 2.0 are shown. The final ranking of biomarkers is based on LDA scores, representing the effect size of each differentially abundant taxon within its respective taxonomic level.

**FIGURE 4:**
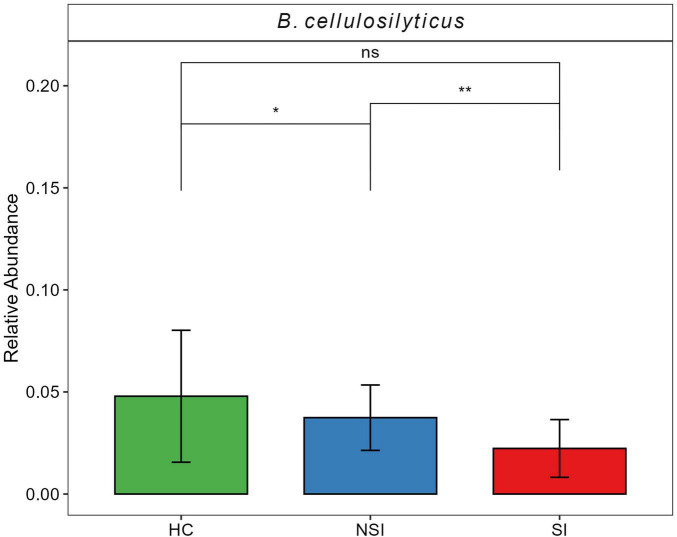
Differential abundance of *Bacteroides cellulosilyticus* across the three groups. Bar plots represent the mean relative abundance of *B. cellulosilyticus* in Healthy Controls (HC, *n* = 52), Non-Suicidal Ideation (NSI, *n* = 32), and Suicidal Ideation (SI, *n* = 57) groups. Error bars indicate the standard error of the mean (SEM). A progressive, step-wise depletion of *B. cellulosilyticus* was observed from the HC to the SI group. Statistical significance was determined using the two-tailed Wilcoxon rank-sum test. ns, not significant; *p < 0.05; **p < 0.01.

LEfSe analysis (Figure 3) identified differentially abundant taxa across multiple taxonomic levels. At the species level, the SI group was significantly enriched in *Clostridium* sp. *AM22 11AC* and *Streptococcus thermophilus*. The NSI group showed enrichment in *Streptococcus pasteurianus*, *Faecalibacillus faecis*, *Methanobrevibacter smithii*, and *Clostridium* sp. *OM07 10AC*. In contrast, the HC group was characterized by *Leuconostoc lactis* and the probiotic species *Bacteroides cellulosilyticus*. At the genus level, the HC group was notably characterized by short-chain fatty acid–producing bacteria, including *Faecalibacterium and Anaerostipes*, while the NSI group was enriched in *GGB1385*. At the phylum level, Candidatus Saccharibacteria was the sole taxon identified with a significant difference, showing specific enrichment in the NSI group. Notably, when using MaAsLin2 (adjusting for age, sex, BMI, and education) with a threshold of *q* < 0.1, no species showed significant differences among groups (Supplementary Figure 1). However, to further validate the clinical relevance of our key findings, we performed a targeted visualization of the relative abundance of *B. cellulosilyticus* (Figure 4). The mean relative abundance (with SEM) revealed a significant, progressive step-wise depletion from HC to NSI, and ultimately to the SI group, confirming its robust downward trend during the development of suicidal ideation. This suggests that while MaAsLin2 may be more conservative due to its compositional adjustments, the biological gradient of key species remains substantial.

**FIGURE 5:**
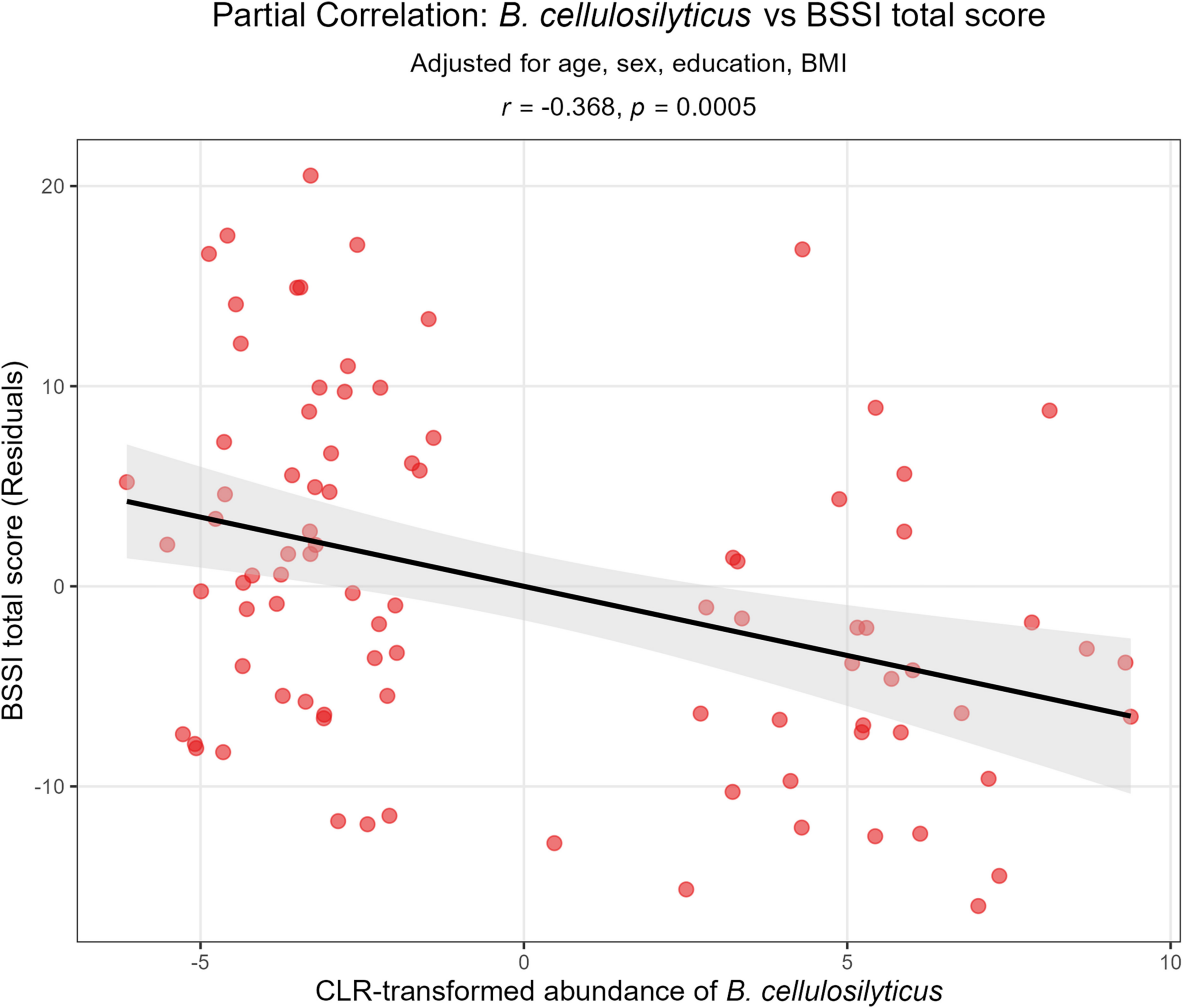
Correlation between Bacteroides cellulosilyticus and suicidal ideation severity. Partial correlation analysis was conducted to evaluate the relationship between the CLR-transformed abundance of *Bacteroides cellulosilyticus* and BSSI total scores. To control for potential confounding factors, age, sex, BMI, and education level were included as covariates in the model. A significant negative correlation was observed (*r* = -0.368, *p* = 5 × 10^– 4^), indicating that a lower abundance of this species is associated with higher suicidal ideation severity. The regression line is shown in black, with the gray shaded area representing the 95% confidence interval. These results were obtained using the ppcor package in R.

*B. cellulosilyticus* was significantly enriched in the HC group. Partial correlation analysis adjusted for covariates showed that its abundance was significantly negatively correlated with BSSI total scores (*r* = -0.368, *p* = 0.0005; Figure 5). Based on these findings, *B. cellulosilyticus* was identified as a key microbial species associated with suicidal ideation.

### 3.4 Differential functional pathways

Based on MaAsLin2 analysis (*q* < 0.1), no significant differences were observed at the KO, KEGG pathway, or CAZy levels between the three groups (HC, SI, and NSI). The comprehensive results for these functional tiers are provided in Supplementary Table 3 and Supplementary Figure 2. However, analysis of MetaCyc pathways revealed that the Peptidoglycan biosynthesis pathway (PWY-5265) was significantly enriched in the NSI group compared to the SI group (*q* < 0.1, Supplementary Figure 3 and Supplementary Table 4). This suggests that the NSI group may have a higher potential for bacterial cell wall component synthesis compared to those with suicidal ideation.

### 3.5 Mediation analysis results

Mediation analysis was conducted with the abundance of the key microbial species as the independent variable, BSSI total score as the dependent variable, and KOs contributed by the key microbial species (taxon-stratified KOs) as mediators.

The mediation models revealed specific molecular pathways through which *B. cellulosilyticus* influences the severity of suicidal ideation. All direct effects were significant (*p* < 0.05), indicating partial mediation effects (Table 2). The total effect of *B. cellulosilyticus* on BSSI scores was also significant.

**TABLE 2:** Significant mediation effects of taxon-stratified KOs between *Bacteroides cellulosilyticus* and BSSI scores in MDD patients.

KO ID: functional description	Pathway/category	Indirect effect	Direct effect	Total effect	p_IE_
K02552: menaquinone-specific isochorismate synthase	Ubiquinone biosynthesis	-0.118	-0.573	-0.691	0.042
K02752: fructose-specific PTS system IIB component	Carbohydrate metabolism	-0.122	-0.569	-0.691	0.03
K02753: beta-glucoside PTS system EIIABC component	Glycolysis/PTS	-0.122	-0.569	-0.691	0.022
K03573: DNA mismatch repair protein MutH	Mismatch repair	-0.126	-0.565	-0.691	0.036
K03632: chromosome partition protein MukB	Cell cycle	-0.117	-0.575	-0.691	0.034
K05306: phosphonoacetaldehyde hydrolase	Phosphonate metabolism	0.159	-0.851	-0.691	0.012
K05590: ATP-dependent RNA helicase SrmB	RNA degradation	-0.112	-0.579	-0.691	0.044
K06204: RNA polymerase-binding transcription factor	Biofilm formation	-0.108	-0.583	-0.691	0.036
K11736: proline-specific permease ProY	Amino acid transport	-0.112	-0.579	-0.691	0.034
K15974: MarR family transcriptional regulator	Drug resistance	-0.132	-0.56	-0.691	0.018
K19416: modulator of FtsH protease	Protein degradation	-0.13	-0.562	-0.691	0.026
K21966: Mat/Ecp fimbriae outer membrane usher protein	Bacterial adhesion	-0.133	-0.558	-0.691	0.016

p_IE_ represents the *p*-value for the Average Causal Mediation Effect (ACME). All models were adjusted for exploratory analysis without correction for multiple comparisons. X, *B. cellulosilyticus*; Y, BSSI total score; M, KO abundance contributed by *B. cellulosilyticus*. Statistical significance is defined as *p* < 0.05.

Specifically, *B. cellulosilyticus* may exert protective effects (i.e., reducing suicidal ideation scores) mainly through pathways related to carbohydrate metabolism (phosphoenolpyruvate:sugar phosphotransferase system, PTS; K02752/K02753), vitamin K2 biosynthesis (K02552), and DNA repair (K03573).

In contrast, K05306 (phosphonoacetaldehyde hydrolase) showed a positive mediation effect, suggesting that it may attenuate the protective role of *B. cellulosilyticus* in suicidal ideation.

## 4 Discussion

This study employed metagenomic sequencing to investigate the association between suicidal ideation and the gut microbiota in MDD patients. The main findings are as follows: (1) *B. cellulosilyticus* was more abundant in HCs and its abundance was significantly negatively correlated with suicidal ideation severity; (2) mediation analysis revealed that this bacterium may potentially negatively regulate suicidal ideation by enhancing pathways related to cellulose degradation-associated transport systems (K02752, K02753) and vitamin K2 biosynthesis (K02552); (3) functional profiling revealed that differences between NSI and SI patients were primarily characterized by alterations in bacterial cell wall biosynthesis potential, specifically the enrichment of the peptidoglycan biosynthesis I (PWY-5265) pathway in the NSI group; and (4) no significant differences in alpha or beta diversity were observed among SI, NSI, and HC groups. Together, these findings suggest a microbiota-metabolite-brain axis underlying suicidality.

Through LEfSe-based differential microbial analysis, *B. cellulosilyticus* was identified as a key discriminative species. To further clarify its clinical relevance beyond its enrichment in HCs, we visualized its distribution across the clinical spectrum, revealing a significant step-wise depletion (HC > NSI > SI) (Figure 4). This downward biological gradient was further supported by partial correlation analysis, showing a significant negative association with BSSI scores.

Notably, while this species did not reach statistical significance under the stringent *q* < 0.1 threshold in the fully adjusted MaAsLin2 model, the model analysis results showed a degree of consistency with the visualized gradient, as the abundance trend remained stably declining in SI. This alignment between the visualized step-wise depletion (HC > NSI > SI) and the consistent direction of effect in the adjusted models suggests that *B. cellulosilyticus* may act as a potential protective-associated factor. Taken together, these findings indicate that *B. cellulosilyticus* should be interpreted as a candidate species of interest, supported by convergent evidence from group comparisons, phenotypic trend visualization, and symptom correlation analyses.

*B. cellulosilyticus* has been described as an “adaptive forager” capable of flexibly utilizing diverse dietary polysaccharides (Kwon et al., 2024; Mu et al., 2025). Its genome encodes the highest number of carbohydrate-active enzymes within the phylum Bacteroidetes, enabling efficient utilization of complex dietary fibers such as xylan and beta-glucan, thereby conferring strong ecological adaptability (McNulty et al., 2013; Robert et al., 2007). Functionally, this bacterium promotes cross-feeding interactions by releasing oligosaccharides that support beneficial microbes such as *Bifidobacterium* (Fernandez-Julia et al., 2023; Wang et al., 2020). In Caco-2 intestinal epithelial cell models, *B. cellulosilyticus* has been shown to reduce the level of interleukin-8 chemokine and transcription of NF-κB, thereby alleviating inflammatory responses. Animal studies further demonstrate its ability to directly regulate host genes associated with neural function, including Gamma-aminobutyric acid, serotonin, and dopamine signaling pathways (Dinić et al., 2025). In addition, its metabolic products extend beyond short-chain fatty acids (SCFAs) (Robert et al., 2007; Wang et al., 2020), such as acetate and propionate, to include neuroprotective metabolites such as hypoxanthine (Mu et al., 2025). Collectively, the overrepresentation of *B. cellulosilyticus* in HCs and its negative correlation with BSSI scores support a coherent interpretation that this species may contribute to resilience against suicidal ideation. Its depletion in MDD patients—particularly those with SI—may reflect a loss of potentially beneficial microbial functions involved in maintaining gut metabolic homeostasis.

To further explore potential mechanisms, we performed mediation analysis. Several KOs involved in carbohydrate transport and utilization, particularly those associated with the phosphotransferase system (PTS; K02752 and K02753), exhibited significant mediation effects. In addition, vitamin K2 (K02552; menaquinone), another mediator identified in our analysis, is an essential cofactor in the electron transport chain and may support efficient energy metabolism in this context. With sufficient substrate uptake and energy supply, carbohydrates (including cellulose and sugars) can be further metabolized into acetate, propionate, and succinate (Robert et al., 2007; Wang et al., 2020), which may represent one plausible pathway linking *B. cellulosilyticus* to host physiology. Given its role as an efficient SCFA producer, we speculate that *B. cellulosilyticus* may influence suicidal ideation by enhancing carbohydrate utilization and downstream metabolite production. However, these findings are based on statistical inference and should be interpreted cautiously, as causal relationships cannot be established in the present study.

Although direct evidence linking SCFAs to suicidal ideation remains limited, indirect evidence suggests a possible connection. For example, higher dietary fiber intake—associated with increased SCFA production—has been linked to a lower risk of suicidal ideation (Huang et al., 2024). SCFAs may influence suicidal ideation through modulation of brain-derived neurotrophic factor (BDNF) and inflammatory processes. Specifically, SCFAs can upregulate BDNF expression via epigenetic mechanisms (Schroeder et al., 2010), while reduced BDNF levels have been associated with increased suicide risk (De Simone et al., 2022). Moreover, SCFAs can decrease intestinal permeability and pH, and exert anti-inflammatory effects through activation of G protein-coupled receptors (Swann et al., 2020). Elevated plasma inflammatory markers, such as C-reactive protein (Park and Kim, 2017) and interleukin-6 (Sun et al., 2023), have also been significantly associated with increased suicidal ideation. These observations provide a biologically plausible framework, although the exact mechanisms remain to be clarified.

Notably, although *B. cellulosilyticus* was negatively correlated with BSSI total scores, mediation analysis identified K05306 (phosphonoacetaldehyde hydrolase) as the only mediator with a positive ACME, suggesting a potential adverse role in suicidality. K05306 catalyzes the hydrolysis of phosphonoacetaldehyde into acetaldehyde and inorganic phosphate and is involved in phosphonate degradation for phosphorus acquisition (Agarwal et al., 2014; Baker et al., 1998). However, its functional role within the gut microbiome remains poorly understood, and the underlying mechanisms require further investigation.

In the functional profiling analysis using MaAsLin2 (*q* < 0.1), the peptidoglycan biosynthesis I pathway (PWY-5265) was identified as significantly depleted in the SI group compared to the NSI group. Although many individual KOs did not reach the stringent significance threshold, several key genes involved in peptidoglycan metabolism, such as K00687 and K12556, showed a strong trend toward enrichment in the NSI group (with *q*-values approaching 0.1). Functional profiling revealed that differences between NSI and SI patients were primarily characterized by alterations in bacterial cell wall biosynthesis potential. Peptidoglycan is an essential structural component of the bacterial cell wall; its synthesis potential is a key indicator of microbial structural integrity and community stability (Gonzalez-Santana and Heijtz, 2020; Wheeler and Gomperts Boneca, 2024). The observed reduction in this pathway suggests that the gut microbiota of SI patients may exhibit diminished stress adaptation and structural robustness.

Notably, no significant differences were observed at the broader KEGG pathway or CAZy (Carbohydrate-Active Enzymes) levels. This lack of global metabolic divergence suggests that the functional shifts associated with suicidality are highly specific rather than reflecting a broad collapse of microbial metabolic capacity. While higher-level metabolic pathways remain largely stable, the observed changes in cell wall biosynthesis pathways may represent a more subtle microbial signature associated with suicidality. However, further studies are required to validate the robustness and specificity of these findings.

Similarly, no significant differences in species-level alpha or beta diversity were observed among the HC, NSI, and SI groups after adjustment for age, sex, and education level, suggesting that suicidality-related microbial alterations may be relatively specific rather than reflecting broad disruption of the gut microbial community. A systematic review of 44 studies reported that alpha diversity is often unchanged in MDD, whereas beta diversity alterations are more commonly observed (Gao et al., 2023; McGuinness et al., 2022). However, studies focusing on suicidality-related phenotypes remain limited and findings are inconsistent. Previous studies have reported altered microbial diversity in suicide-related populations. For example, suicide decedents showed significant beta diversity separation from healthy controls (Kozhakhmetov et al., 2025), while MDD patients with suicidal ideation or recent suicide attempts exhibited distinct beta diversity patterns compared with non-suicidal patients (Chen and Wu, 2025; Prandovszky et al., 2025). The discrepancies between our findings and previous reports may be attributable to differences in suicide-related phenotypes, assessment tools, covariate adjustment strategies, as many previous studies did not fully control for potential confounders. Additional methodological factors, including sample size, sequencing approaches and depth (e.g., 16S rRNA sequencing versus shotgun metagenomics), medication exposure, dietary patterns, and geographic background, may also contribute to inconsistent findings across studies.

This study has several strengths. First, we performed a comprehensive analysis of gut microbiota composition and diversity across three comparison groups, using metagenomic sequencing to obtain high-resolution taxonomic and functional profiles, which enhanced the accuracy of the results and enabled mechanistic exploration. Second, standardized and validated diagnostic interviews and mood assessment tools administered by trained professionals were used, ensuring the reliability of clinical phenotyping. Third, all patients with MDD were drug-naïve at first episode, minimizing the potential confounding effects of antidepressant treatment on the gut microbiota. Most analyses adjusted for key covariates, including age, sex, educational level, and BMI, to improve the robustness of the findings, with the exception of the LEfSe analysis.

Several limitations should be acknowledged. First, the sample size imbalance among groups (HC, SI, and NSI) may have limited the statistical power to detect subtler microbial signatures, particularly within the NSI group. Second, while *B. cellulosilyticus* showed a significant correlation with BSSI scores, it did not reach the significance threshold in the MaAsLin2 analysis after adjusting for all covariates (age, sex, BMI, and education). This suggests that while a notable association exists, the potential of this bacterium as a standalone biomarker for SI should be interpreted with caution. In addition, given the exploratory nature of this study, mediation analyses were not corrected for multiple testing (e.g., FDR), which may increase the risk of false-positive findings. Third, while we controlled for several key demographic variables, the potential for residual confounding from unmeasured factors, such as detailed dietary habits, cannot be entirely ruled out. Fourth, the compositional nature of metagenomic data means that observed changes reflect relative abundances, which warrants cautious interpretation compared to absolute quantification. Fifth, the cross-sectional design precludes any inference of causality between gut microbiota and suicidal ideation. Finally, the functional pathways identified are based on metagenomic inference (genomic potential) rather than active metabolic expression. Although we speculate that SCFAs are key mediators through which *B. cellulosilyticus* influences suicidality, SCFAs were not directly measured. Therefore, these findings require further validation using complementary approaches, such as serum and fecal metabolomics.

## 5 Conclusion

Investigating the composition and functional characteristics of the gut microbiome may provide novel insights into the prevention and intervention of suicidal ideation. In the present study, *B. cellulosilyticus* was identified as a microbial species associated with the severity of suicidal ideation in MDD patients, showing a negative correlation with symptom severity. Although the underlying mechanisms remain to be fully elucidated, this association may be partly linked to its involvement in microbial metabolic processes, including the production of SCFAs. Notably, despite its potential relevance, this bacterium remains relatively understudied. Overall, our findings suggest that alterations in specific gut microbial taxa and their metabolic functions may be involved in the development of suicidal ideation. These results provide a framework for future studies to further explore microbiome-based mechanisms and potential intervention strategies.

## Data Availability

The datasets generated and analyzed during the current study are not publicly available due to institutional and ethical restrictions related to data security and participant confidentiality. Datasets are available from the corresponding author upon reasonable request.

## References

[B1] Agarwal V. Peck S. C. Chen J. H. Borisova S. A. Chekan J. R. van der Donk W. A. et al. (2014). Structure and function of phosphonoacetaldehyde dehydrogenase: the missing link in phosphonoacetate formation. *Chem. Biol.* 21 125–135. 10.1016/j.chembiol.2013.11.006 24361046 PMC4313731

[B2] Ahrens A. P. Sanchez-Padilla D. E. Drew J. C. Oli M. W. Roesch L. F. W. Triplett E. W. (2022). Saliva microbiome, dietary, and genetic markers are associated with suicidal ideation in university students. *Sci. Rep.* 12:14306. 10.1038/s41598-022-18020-2 35995968 PMC9395396

[B3] Baker A. S. Ciocci M. J. Metcalf W. W. Kim J. Babbitt P. C. Wanner B. L. et al. (1998). Insights into the mechanism of catalysis by the P-C bond-cleaving enzyme phosphonoacetaldehyde hydrolase derived from gene sequence analysis and mutagenesis. *Biochemistry* 37 9305–9315. 10.1021/bi972677d 9649311

[B4] Cai H. Jin Y. Liu S. Zhang Q. Zhang L. Cheung T. et al. (2021). Prevalence of suicidal ideation and planning in patients with major depressive disorder: a meta-analysis of observation studies. *J. Affect. Disord.* 293 148–158. 10.1016/j.jad.2021.05.115 34192629

[B5] Cai L. F. Wang S. B. Hou C. L. Li Z. B. Liao Y. J. Jia F. J. (2022). Association between non-suicidal self-injury and gut microbial characteristics in Chinese adolescent. *Neuropsychiatr. Dis. Treat.* 18 1315–1328. 10.2147/NDT.S360588 35799798 PMC9255420

[B6] Carter G. Spittal M. J. (2018). Suicide risk assessment. *Crisis* 39 229–234. 10.1027/0227-5910/a000558 29972324

[B7] Chen B. Huang Y. Feng S. Liu C. Li H. Zhang Z. et al. (2026). Correlation between cognitive dysfunction and amygdala connectivity in first-episode, treatment-naïve patients with anxious major depressive disorder. *J. Psychiatr. Res.* 197, 210–218. 10.1016/j.jpsychires.2026.02.052 41797253

[B8] Chen S. Zhu B. Lu X. Huang Y. Wang S. Wang W. et al. (2026). Integrative multi-kingdom gut microbiome analysis uncovers clinical signatures of major depressive disorder. *J. Affect. Disord.* 408:121858. 10.1016/j.jad.2026.121858 42035799

[B9] Chen V. C. H. Wu S. I. (2025). An exploratory analysis on the association between suicidal ideation and the microbiome in patients with or without major depressive disorder. *J. Affect. Disord.* 370 362–372. 10.1016/j.jad.2024.10.120 39481689

[B10] De Simone S. Bosco M. A. La Russa R. Vittorio S. Di Fazio N. Neri M. et al. (2022). Suicide and neurotrophin factors: a systematic review of the correlation between BDNF and GDNF and self-killing. *Healthcare* 11:78. 10.3390/healthcare11010078 36611538 PMC9818650

[B11] Dickerson F. Adamos M. Katsafanas E. Khushalani S. Origoni A. Savage C. et al. (2017). The association between immune markers and recent suicide attempts in patients with serious mental illness: a pilot study. *Psychiatry Res.* 255 8–12. 10.1016/j.psychres.2017.05.005 28505469

[B12] Dinić M. ?okić J. Jakovljević S. Brdarić E. Mitrović H. Bisenić A. et al. (2025). Insight into immunoregulatory and neuromodulatory capability of *Bacteroides cellulosilyticus* and *Bacteroides xylanisolvens* human gut microbiota isolates. *Sci. Rep.* 15:38058. 10.1038/s41598-025-21839-0 41168250 PMC12575647

[B13] Dong M. Wang S. B. Li Y. Xu D. D. Ungvari G. S. Ng C. H. et al. (2018). Prevalence of suicidal behaviors in patients with major depressive disorder in China: a comprehensive meta-analysis. *J. Affect. Disord.* 225 32–39. 10.1016/j.jad.2017.07.043 28779680

[B14] Fernandez-Julia P. Black G. W. Cheung W. Van Sinderen D. Munoz-Munoz J. (2023). Fungal β-glucan-facilitated cross-feeding activities between *Bacteroides* and *Bifidobacterium species*. *Commun. Biol.* 6:576. 10.1038/s42003-023-04970-4 37253778 PMC10229575

[B15] Gao M. Wang J. Liu P. Tu H. Zhang R. Zhang Y. et al. (2023). Gut microbiota composition in depressive disorder: a systematic review, meta-analysis, and meta-regression. *Transl. Psychiatry* 13:379. 10.1038/s41398-023-02670-5 38065935 PMC10709466

[B16] García-Gutiérrez M. S. Caparros E. Torregrosa A. B. Martínez-López S. Gasparyan A. Giner S. et al. (2025). Alterations of pro- and anti-inflammatory cytokines balance and superoxide dismutase in the dorsolateral prefrontal cortex of suicide decedents. *Neurotherapeutics* 22:e00634. 10.1016/j.neurot.2025.e00634 40615287 PMC12491799

[B17] Geoffroy P. A. Oquendo M. A. Courtet P. Blanco C. Olfson M. Peyre H. et al. (2021). Sleep complaints are associated with increased suicide risk independently of psychiatric disorders: results from a national 3-year prospective study. *Mol. Psychiatry* 26 2126–2136. 10.1038/s41380-020-0735-3 32355334

[B18] Gonzalez-Santana A. Heijtz R. D. (2020). Bacterial peptidoglycans from microbiota in neurodevelopment and behavior. *Trends Mol. Med.* 26 729–743. 10.1016/j.molmed.2020.05.003 32507655

[B19] Hawton K. van Heeringen K. (2009). Suicide. *Lancet* 373 1372–1381. 10.1016/S0140-6736(09)60372-X 19376453

[B20] He L. Huang L. Huang Y. Li H. Zhang Z. Li J. et al. (2025). Prevalence and influencing factors of anxiety, depression, and burnout among teachers in China: a cross-sectional study. *Front. Psychiatry* 16:1567553. 10.3389/fpsyt.2025.1567553 40182199 PMC11965650

[B21] He Y. Wu F. Zhang Z. Yi Y. Feng S. Lin K. et al. (2025). Association between EEG microstate and cognitive function in depressed patients with and without suicidal ideation. *BMC Psychiatry* 26:60. 10.1186/s12888-025-07617-2 41257797 PMC12829176

[B22] Huang H. Fu J. Lu K. Fu Y. Zhuge P. Yao Y. (2024). Association between dietary fiber intake and suicidal ideation: a cross-sectional survey. *Front. Nutr.* 11:1465736. 10.3389/fnut.2024.1465736 39539370 PMC11557476

[B23] Huang Y. Wang Y. Wang H. Liu Z. Yu X. Yan J. et al. (2019). Prevalence of mental disorders in China: a cross-sectional epidemiological study. *Lancet Psychiatry* 6 211–224. 10.1016/S2215-0366(18)30511-X 30792114

[B24] Kang S. G. Lee J. H. Lee K. Kim H. C. Seo W. S. Won S. (2020). The rs6265 polymorphism of the BDNF gene is related to higher-lethality suicide attempts in the korean population. *Psychiatry Investig.* 17 417–423. 10.30773/pi.2020.0012 32295326 PMC7265018

[B25] Kara F. Gülbahçe A. (2024). “The Relationship between Depression and Suicide,” in *The Association Between Depression and Suicidal Behavior*, ed. Manuel Morales-Rodríguez F. (London: IntechOpen), 10.5772/intechopen.1006685

[B26] Kozhakhmetov S. Kossumov A. Zhakupova T. Polyakova T. Imambayeva N. Syzdykova B. et al. (2025). Characterization of gut microbiome composition in depression and completed suicide. *Int. J. Mol. Sci.* 26:4880. 10.3390/ijms26104880 40430019 PMC12112742

[B27] Kwon H. Nam E. H. Kim H. Jo H. Bang W. Y. Lee M. et al. (2024). Effect of *Lacticaseibacillus rhamnosus* IDCC 3201 on irritable bowel syndrome with constipation: a randomized, double-blind, and placebo-controlled trial. *Sci. Rep.* 14:22384. 10.1038/s41598-024-72887-x 39333245 PMC11437119

[B28] Li K. Lyu H. Zhang L. Ma S. Wang K. Fu Y. et al. (2025). Association between dietary patterns and suicide ideation among depressed adults: insights from NHANES 2007-2020. *J. Affect. Disord.* 377 235–244. 10.1016/j.jad.2025.02.073 39988135

[B29] Li Y. Zhu B. Wu X. Meng L. Huang S. Wu K. (2026). Microbiota-immune crosstalk underlying cancer-induced depression in patients with breast cancer. *J. Affect. Disord.* 407:121694. 10.1016/j.jad.2026.121694 41905614

[B30] Lindqvist D. Janelidze S. Hagell P. Erhardt S. Samuelsson M. Minthon L. et al. (2009). Interleukin-6 is elevated in the cerebrospinal fluid of suicide attempters and related to symptom severity. *Biol. Psychiatry* 66 287–292. 10.1016/j.biopsych.2009.01.030 19268915

[B31] Maes M. Zhou B. Vasupanrajit A. Jirakran K. Klomkliew P. Chanchaem P. et al. (2024). A further examination of growth factors, T helper 1 polarization, and the gut microbiome in major depression: associations with reoccurrence of illness, cognitive functions, suicidal behaviors, and quality of life. *J. Psychiatr. Res.* 176 430–441. 10.1016/j.jpsychires.2024.06.037 38968876

[B32] Mallick H. Rahnavard A. McIver L. J. Ma S. Zhang Y. Nguyen L. H. et al. (2021). Multivariable association discovery in population-scale meta-omics studies. *PLoS Comput. Biol.* 17:e1009442. 10.1371/journal.pcbi.1009442 34784344 PMC8714082

[B33] McGuinness A. J. Davis J. A. Dawson S. L. Loughman A. Collier F. O’Hely M. et al. (2022). A systematic review of gut microbiota composition in observational studies of major depressive disorder, bipolar disorder and schizophrenia. *Mol. Psychiatry* 27 1920–1935. 10.1038/s41380-022-01456-3 35194166 PMC9126816

[B34] McNulty N. P. Wu M. Erickson A. R. Pan C. Erickson B. K. Martens E. C. et al. (2013). Effects of diet on resource utilization by a model human gut microbiota containing *Bacteroides cellulosilyticus* WH2, a symbiont with an extensive glycobiome. *PLoS Biol.* 11:e1001637. 10.1371/journal.pbio.1001637 23976882 PMC3747994

[B35] Milton A. C. Davenport T. A. Iorfino F. Flego A. Burns J. M. Hickie I. B. (2020). Suicidal thoughts and behaviors and their associations with transitional life events in men and women: findings from an international web-based sample. *JMIR Ment. Health* 7:e18383. 10.2196/18383 32915160 PMC7519425

[B36] Morena D. Lippi M. Scopetti M. Turillazzi E. Fineschi V. (2025). Leaky gut biomarkers as predictors of depression and suicidal risk: a systematic review and meta-analysis. *Diagnostics* 15:1683. 10.3390/diagnostics15131683 40647682 PMC12249198

[B37] Mu M. Xu Q. Hao Q. Li X. Wu Z. Zhang Q. et al. (2025). Multiomics analysis of *Bacteroides cellulosilyticus* anticolitis via gut microbiota metabolite-mediated PI3K-Akt signaling pathway. *J. Agric. Food Chem.* 73 16333–16347. 10.1021/acs.jafc.5c00637 40539526

[B38] Neupane S. P. Daray F. M. Ballard, Galfalvy H. Itzhaky L. Segev A. et al. (2023). Immune-related biomarkers and suicidal behaviors: a meta-analysis. *Eur. Neuropsychopharmacol.* 75 15–30. 10.1016/j.euroneuro.2023.05.009 37356288

[B39] O’Connor R. C. Nock M. K. (2014). The psychology of suicidal behaviour. *Lancet Psychiatry* 1 73–85. 10.1016/S2215-0366(14)70222-6 26360404

[B40] Ohlsson L. Gustafsson A. Lavant E. Suneson K. Brundin L. Westrin Å et al. (2019). Leaky gut biomarkers in depression and suicidal behavior. *Acta Psychiatr. Scand.* 139 185–193. 10.1111/acps.12978 30347427 PMC6587489

[B41] Park R. J. Kim Y. H. (2017). Association between high sensitivity CRP and suicidal ideation in the Korean general population. *Eur. Neuropsychopharmacol.* 27 885–891. 10.1016/j.euroneuro.2017.06.010 28663123

[B42] Prandovszky E. Liu H. Severance E. G. Splan V. W. Dickerson F. B. Yolken R. H. (2025). Altered gut microbial diversity, composition, and metabolomic potential in patients with major depressive disorder and recent suicide attempt. *Brain Behav. Immun. Health* 48:101081. 10.1016/j.bbih.2025.101081 40896414 PMC12390952

[B43] Robert C. Chassard C. Lawson P. A. Bernalier-Donadille A. (2007). *Bacteroides cellulosilyticus* sp. nov., a cellulolytic bacterium from the human gut microbial community. *Int. J. Syst. Evol. Microbiol.* 57(Pt 7), 1516–1520. 10.1099/ijs.0.64998-0 17625186

[B44] Schroeder M. Krebs M. O. Bleich S. Frieling H. (2010). Epigenetics and depression: current challenges and new therapeutic options. *Curr. Opin. Psychiatry* 23 588–592. 10.1097/YCO.0b013e32833d16c1 20644477

[B45] Sha Q. Fu Z. Escobar Galvis M. L. Madaj Z. Underwood M. D. Steiner J. A. et al. (2024). Integrative transcriptome- and DNA methylation analysis of brain tissue from the temporal pole in suicide decedents and their controls. *Mol. Psychiatry* 29 134–145. 10.1038/s41380-023-02311-9 37938766 PMC11078738

[B46] Sudol K. Mann J. J. (2017). Biomarkers of suicide attempt behavior: towards a biological model of risk. *Curr. Psychiatry Rep.* 19:31. 10.1007/s11920-017-0781-y 28470485

[B47] Sun S. Wilson C. M. Alter S. Ge Y. Hazlett E. A. Goodman M. et al. (2023). Association of interleukin-6 with suicidal ideation in veterans: a longitudinal perspective. *Front. Psychiatry* 14:1231031. 10.3389/fpsyt.2023.1231031 37779624 PMC10540304

[B48] Swann O. G. Kilpatrick M. Breslin M. Oddy W. H. (2020). Dietary fiber and its associations with depression and inflammation. *Nutr. Rev.* 78 394–411. 10.1093/nutrit/nuz072 31750916

[B49] Thompson D. S. Fowler J. C. Bradshaw M. R. Frueh B. C. Weinstein B. L. Petrosino J. et al. (2021). Is the gut microbiota associated with suicidality? Non-significant finding among a large cohort of psychiatrically hospitalized individuals with serious mental illness. *J. Affect. Disord. Rep.* 6:100266. 10.1016/j.jadr.2021.100266

[B50] Wang M. Kong Y. Chen Y. Lei Z. Lin Q. Zhou R. et al. (2025). How does deliberate rumination influence the association between stigma and suicide ideation in depressed patients: a mediation analysis. *Int. J. Ment. Health Nurs.* 34:e70065. 10.1111/inm.70065 40437695

[B51] Wang Y. Shao S. Guo C. Zhang S. Li M. Ding K. (2020). The homogenous polysaccharide SY01-23 purified from leaf of *Morus alba* L. has bioactivity on human gut *Bacteroides ovatus* and *Bacteroides cellulosilyticus*. *Int. J. Biol. Macromol.* 158 698–707. 10.1016/j.ijbiomac.2020.05.009 32387599

[B52] Wheeler R. Gomperts Boneca I. (2024). The hidden base of the iceberg: gut peptidoglycome dynamics is foundational to its influence on the host. *Gut Microbes* 16:2395099. 10.1080/19490976.2024.2395099 39239828 PMC11382707

[B53] World Health Organization [WHO] (2026). *World Health Organization Regional Office for the Eastern Mediterranean. Depression: Introduction.* Geneva: WHO.

[B54] Wu F. Zhu B. Feng S. Li H. Zhou J. Ning Y. et al. (2026). The Brain-Gut Health Initiative (BIGHI): a prospective cohort on psychiatric disorders in China. *Research* 9:1142. 10.34133/research.1142 41783048 PMC12953926

[B55] Wu J. Huang Y. Lu H. Li H. Zhou S. Zhang Z. et al. (2026). Effects of serum hypersensitive C-reactive protein and BMI on cognitive dysfunction in first-episode and drug-naive patients with major depressive disorder. *BMC Psychiatry* 26:178. 10.1186/s12888-026-07782-y 41593562 PMC12918109

[B56] Xu S. Xiong J. Qin X. Ma M. Peng Y. Cheng J. et al. (2025). Association between gut microbiota and perinatal depression and anxiety among a pregnancy cohort in Hunan, China. *Brain Behav. Immun.* 125 168–177. 10.1016/j.bbi.2024.12.150 39736365

[B57] Zhang Z. Li H. Huang Y. He Y. Xu W. Wu J. et al. (2026). EEG microstate dynamics reveal progressive sensorimotor network dysfunction across levels of anxiety severity in drug-naïve major depressive disorder. *J. Affect. Disord.* 402:121373. 10.1016/j.jad.2026.121373 41679393

[B58] Zhu B. Chen S. Diao Y. Wang W. Huang Y. Liang L. et al. (2026). Dissecting the ecological structure of health and disease in the global gut microbiome. *Adv. Sci.* 10.1002/advs.202517087 42153961 PMC13335992

