# Supplementary material for: Novel insights into gut microbiota alterations in major depressive disorder with suicidal ideation: a metagenomic analysis
Source: Front Microbiol. 2026 Jun 10;17:1843301. doi: 10.3389/fmicb.2026.1843301 (PMC13290911; doi:10.3389/fmicb.2026.1843301)
Supplement: Supplementary file 1 [file Supplementary_file_1.zip › Supplementary Figures S1-S3.DOCX]

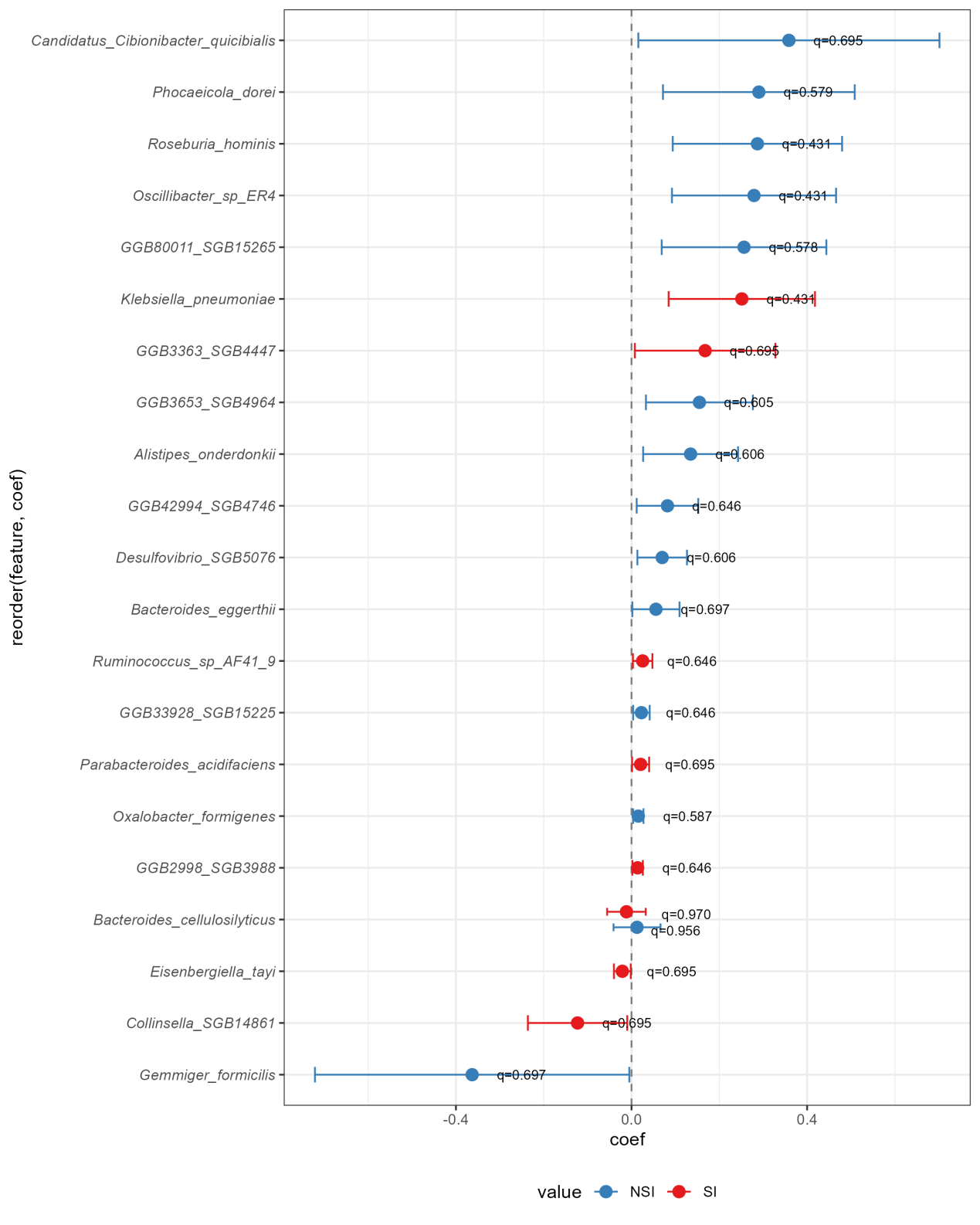


**Supplementary Figure S1**. MaAsLin2 associations between microbial species and study groups.

The forest plot displays the association coefficients (with 95% confidence intervals) for the top 20 microbial species ranked by *q*-value, along with *Bacteroides cellulosilyticus*. Blue points/lines represent the Non-suicidal Ideation (NSI) group, and red represents the Suicidal Ideation (SI) group, both compared against the Healthy Control (HC) group as the reference. Although these species did not reach the strict significance threshold (*q* < 0.1), they represent the most prominent taxonomic trends. Linear models were adjusted for age, sex, BMI, and education level. *q*-values were calculated using the Benjamini-Hochberg method.


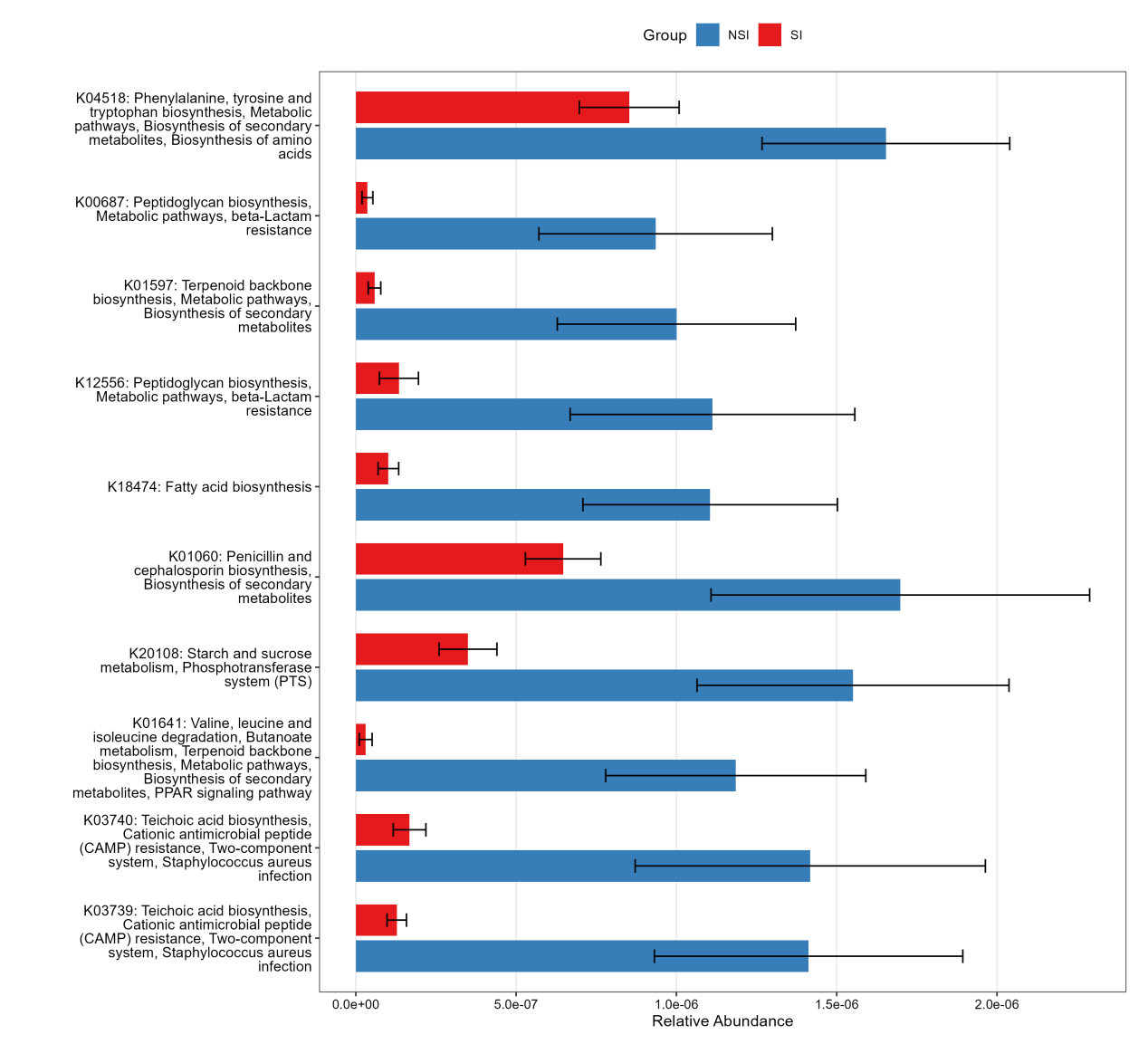


**Supplementary Figure S2**. Distribution of nominal differential KEGG Orthology (KO) groups across SI and NSI groups.

Comparison of relative abundances for the top KO identifiers with exploratory significance (*q* < 0.25). Bars represent the mean relative abundance (±SEM) for the NSI (blue) and SI (red) groups. These functional features involve pathways such as peptidoglycan biosynthesis, fatty acid biosynthesis, and teichoic acid biosynthesis. Statistical associations were determined using MaAsLin2 linear models adjusted for age, sex, BMI, and education level. Note that while these features show visible trends, they did not meet the conservative FDR threshold of 0.1.


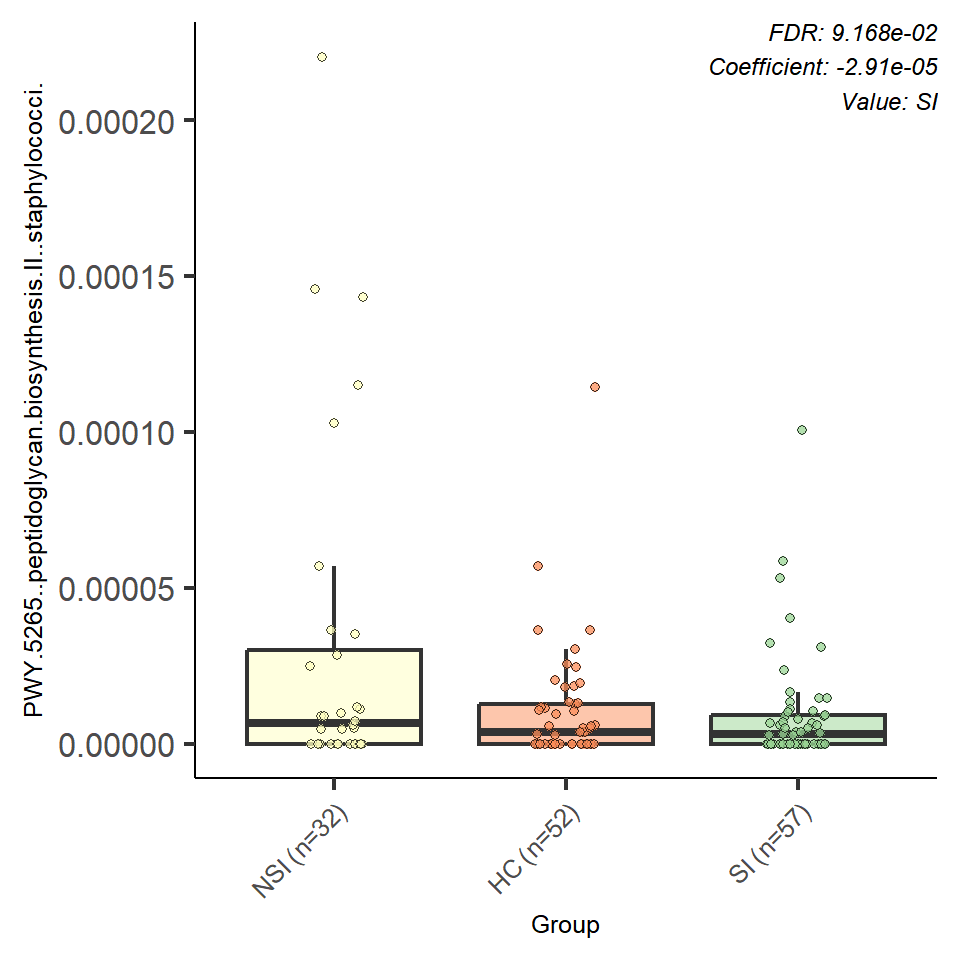


**Supplementary Figure S3**. Significant enrichment of the Peptidoglycan biosynthesis pathway (PWY-5265) in the NSI group.

Box plots illustrate the relative abundance of the MetaCyc pathway PWY-5265 (Peptidoglycan biosynthesis II; staphylococci) across NSI (n=32), HC (n=52), and SI (n=57) groups. The NSI group exhibited a significantly higher abundance of this pathway compared to the SI group (FDR = 9.168×10^-2^, Coefficient = -2.91×10^-5^ with SI as the target and NSI as the reference). The horizontal line within the box represents the median, and the whiskers indicate the interquartile range. The model was adjusted for age, sex, BMI, and education level.
