# Supplementary material for: Novel insights into gut microbiota alterations in major depressive disorder with suicidal ideation: a metagenomic analysis
Source: Front Microbiol. 2026 Jun 10;17:1843301. doi: 10.3389/fmicb.2026.1843301 (PMC13290911; doi:10.3389/fmicb.2026.1843301)
Supplement: Supplementary file 1 [file Supplementary_file_1.zip › Supplementary material 1.DOCX]

Supplementary file 1

Metagenomic Sequencing and Data Quality Control

Total genomic DNA was randomly fragmented to a size of approximately 350 bp using a Covaris ultrasonicator. The fragmented DNA underwent end-repair, addition of a 3’ adenine (A) tail, and ligation with Illumina sequencing adapters. Size selection of the adapter-ligated libraries was performed using Agencourt SPRIselect beads (Beckman Coulter, USA) to ensure optimal insert size. The purified libraries were then enriched by PCR amplification using high-fidelity polymerases.

Library quality and insert sizes were assessed using the Agilent 5400 system (AATI), and the effective concentration was precisely determined via qPCR (1.5 nM). Qualified libraries were pooled and sequenced on the Illumina NovaSeq platform (Illumina Inc., USA) using a paired-end 150 bp (PE150) strategy.

Raw sequencing data (FASTQ format) were subjected to rigorous quality control using fastp software. The filtering process included: (1) removal of adapter-contaminated read pairs; (2) removal of reads containing more than 10 bp of ambiguous "N" bases; and (3) removal of low-quality reads (where >50% of bases had a quality score Q ≤ 5).
