# Supplementary material for: Novel insights into gut microbiota alterations in major depressive disorder with suicidal ideation: a metagenomic analysis
Source: Front Microbiol. 2026 Jun 10;17:1843301. doi: 10.3389/fmicb.2026.1843301 (PMC13290911; doi:10.3389/fmicb.2026.1843301)
Supplement: Supplementary file 1 [file Supplementary_file_1.zip › Supplementary Table 1.docx]

**Supplementary Table 1**. Linear model (Type III Sum of Squares) analysis of alpha diversity indices among the HC, NSI, and SI groups

| **Metric** | **Term** | **Sum Sq** | **Df** | ***F* value** | ***p*** |
| --- | --- | --- | --- | --- | --- |
| Shannon | (Intercept) | 20.215 | 1 | 138.098 | <0.001 |
| Shannon | age | 0.362 | 1 | 2.475 | 0.118 |
| Shannon | sex | 0.334 | 1 | 2.285 | 0.133 |
| Shannon | education | 0.443 | 1 | 3.029 | 0.084 |
| Shannon | BMI | 0.022 | 1 | 0.151 | 0.698 |
| Shannon | Group | 0.049 | 2 | 0.168 | 0.845 |
| Shannon | Residuals | 19.615 | 134 | NA | NA |
| Simpson | (Intercept) | 1.593 | 1 | 908.667 | <0.001 |
| Simpson | age | 0.004 | 1 | 2.097 | 0.150 |
| Simpson | sex | 0.003 | 1 | 1.966 | 0.163 |
| Simpson | education | 0.007 | 1 | 3.833 | 0.052 |
| Simpson | BMI | 0.001 | 1 | 0.291 | 0.591 |
| Simpson | Group | 0.001 | 2 | 0.385 | 0.681 |
| Simpson | Residuals | 0.235 | 134 | NA | NA |
| Observed | (Intercept) | 21751.326 | 1 | 20.965 | <0.001 |
| Observed | age | 828.772 | 1 | 0.799 | 0.373 |
| Observed | sex | 587.042 | 1 | 0.566 | 0.453 |
| Observed | education | 1666.028 | 1 | 1.606 | 0.207 |
| Observed | BMI | 324.555 | 1 | 0.313 | 0.577 |
| Observed | Group | 258.010 | 2 | 0.124 | 0.883 |
| Observed | Residuals | 139027.688 | 134 | NA | NA |

Note: Alpha diversity indices, including Observed richness, Shannon index, and Simpson index, were calculated using the R package vegan (version 2.6–4). Differences among the HC, NSI, and SI groups were assessed using linear models (LM) with Type III Sum of Squares. Age, sex, BMI, and education level were incorporated into the models as covariates to adjust for potential confounding factors. Sum Sq, Sum of Squares; Df, Degrees of freedom; NA, not applicable. A *p*-value < 0.05 was considered statistically significant.
