# Supplementary material for: Novel insights into gut microbiota alterations in major depressive disorder with suicidal ideation: a metagenomic analysis
Source: Front Microbiol. 2026 Jun 10;17:1843301. doi: 10.3389/fmicb.2026.1843301 (PMC13290911; doi:10.3389/fmicb.2026.1843301)
Supplement: Supplementary file 1 [file Supplementary_file_1.zip › Supplementary Table 2.docx]

**Supplementary Table 2.** Permutational multivariate analysis of variance (PERMANOVA) based on Bray–Curtis distances among the HC, NSI, and SI groups.

| **Variable** | **Df** | **Sum Sq** | ***R*^2^** | ***F*** | ***p*** |
| --- | --- | --- | --- | --- | --- |
| age | 1 | 0.285 | 0.007 | 1.051 | 0.389 |
| sex | 1 | 0.449 | 0.012 | 1.657 | 0.015 |
| education | 1 | 0.293 | 0.008 | 1.079 | 0.326 |
| BMI | 1 | 0.411 | 0.011 | 1.515 | 0.035 |
| Group | 2 | 0.552 | 0.014 | 1.019 | 0.397 |
| Residuals | 134 | 36.319 | 0.946 | NA | NA |
| Total | 140 | 38.385 | 1.000 | NA | NA |

Note: PERMANOVA was performed using the adonis2 function in the R package vegan with 999 permutations based on Bray–Curtis distances. Age, sex, BMI, and education level were incorporated into the model to control for demographic and clinical confounders. Df, degrees of freedom; Sum Sq, sum of squares; *R*², effect size representing the proportion of variance explained; *F*, *F* statistic by permutation; *p*, permutation *p*-value; NA, not applicable. A *p*-value < 0.05 was considered statistically significant.
