# Supplementary material for: Novel insights into gut microbiota alterations in major depressive disorder with suicidal ideation: a metagenomic analysis
Source: Front Microbiol. 2026 Jun 10;17:1843301. doi: 10.3389/fmicb.2026.1843301 (PMC13290911; doi:10.3389/fmicb.2026.1843301)
Supplement: Supplementary file 1 [file Supplementary_file_1.zip › Supplementary Table 3.docx]

**SupplementaryTable 3.** Differential functional analysis of microbial features among HC, SI, and NSI groups using MaAsLin2.

| functionalpathway | reference | feature | value | coef | stderr | N.not.0 | *p* | *q* |
| --- | --- | --- | --- | --- | --- | --- | --- | --- |
| KO | HC | K08717 | NSI | 0.000004 | 0.000001 | 64 | 0.0002 | 0.1013 |
| KO | HC | K08364 | NSI | 0.000004 | 0.000001 | 72 | 0.0009 | 0.2233 |
| KO | HC | K00348 | NSI | 0.000003 | 0.000001 | 118 | 0.0010 | 0.2308 |
| KO | HC | K11381 | NSI | 0.000004 | 0.000001 | 68 | 0.0012 | 0.2363 |
| KO | NSI | K08717 | HC | -0.000004 | 0.000001 | 64 | 0.0002 | 0.1013 |
| KO | NSI | K03713 | SI | -0.000002 | 0.000001 | 30 | 0.0004 | 0.1389 |
| KO | NSI | K07118 | SI | -0.000003 | 0.000001 | 34 | 0.0004 | 0.1389 |
| KO | NSI | K08717 | SI | -0.000003 | 0.000001 | 64 | 0.0005 | 0.1546 |
| KO | NSI | K03740 | SI | -0.000002 | 0.000001 | 66 | 0.0007 | 0.1697 |
| KO | NSI | K08987 | SI | -0.000002 | 0.000001 | 74 | 0.0006 | 0.1697 |
| KO | NSI | K20107 | SI | -0.000002 | 0.000001 | 84 | 0.0006 | 0.1697 |
| KO | NSI | K20108 | SI | -0.000002 | 0.000001 | 84 | 0.0006 | 0.1697 |
| KO | NSI | K21681 | SI | -0.000004 | 0.000001 | 67 | 0.0007 | 0.1766 |
| KO | NSI | K02243 | SI | -0.000002 | 0.000001 | 49 | 0.0007 | 0.1823 |
| KO | NSI | K01641 | SI | -0.000002 | 0.000001 | 19 | 0.0008 | 0.1902 |
| KO | NSI | K03492 | SI | -0.000002 | 0.000001 | 21 | 0.0010 | 0.2045 |
| KO | NSI | K08364 | HC | -0.000004 | 0.000001 | 72 | 0.0009 | 0.2045 |
| KO | NSI | K01060 | SI | -0.000002 | 0.000001 | 100 | 0.0010 | 0.2062 |
| KO | NSI | K03739 | SI | -0.000002 | 0.000001 | 58 | 0.0010 | 0.2062 |
| KO | NSI | K00348 | HC | -0.000003 | 0.000001 | 118 | 0.0010 | 0.2077 |
| KO | NSI | K18474 | SI | -0.000002 | 0.000001 | 49 | 0.0011 | 0.2125 |
| KO | NSI | K11381 | HC | -0.000004 | 0.000001 | 68 | 0.0012 | 0.2130 |
| KO | NSI | K18349 | HC | -0.000006 | 0.000002 | 138 | 0.0013 | 0.2280 |
| KO | NSI | K02530 | SI | -0.000002 | 0.000001 | 72 | 0.0014 | 0.2324 |
| KO | NSI | K07138 | HC | -0.000004 | 0.000001 | 109 | 0.0014 | 0.2332 |
| KO | NSI | K12556 | SI | -0.000002 | 0.000001 | 26 | 0.0015 | 0.2332 |
| KO | NSI | K01597 | SI | -0.000002 | 0.000001 | 45 | 0.0015 | 0.2422 |
| KO | NSI | K06989 | HC | -0.000003 | 0.000001 | 88 | 0.0016 | 0.2464 |
| KO | NSI | K22212 | SI | -0.000003 | 0.000001 | 73 | 0.0016 | 0.2464 |
| KO | NSI | K02245 | HC | -0.000003 | 0.000001 | 86 | 0.0017 | 0.2470 |
| KEGG | HC | General.function.prediction.only | NSI | -0.512241 | 0.130396 | 141 | 0.0001 | 0.1215 |
| KEGG | HC | Mitophagy...animal | NSI | 1.280579 | 0.327581 | 38 | 0.0001 | 0.1215 |
| KEGG | HC | Amino.acid.metabolism | NSI | -0.427583 | 0.150508 | 141 | 0.0052 | 0.2437 |
| KEGG | HC | Aminobenzoate.degradation | NSI | -0.616864 | 0.187207 | 141 | 0.0013 | 0.2437 |
| KEGG | HC | Apelin.signaling.pathway | NSI | 1.002198 | 0.351418 | 25 | 0.0050 | 0.2437 |
| KEGG | HC | Arrhythmogenic.right.ventricular.cardiomyopathy | NSI | 0.513880 | 0.165530 | 27 | 0.0023 | 0.2437 |
| KEGG | HC | Autophagy...animal | NSI | 1.052345 | 0.370276 | 31 | 0.0052 | 0.2437 |
| KEGG | HC | Biofilm.formation...Vibrio.cholerae | NSI | -0.386357 | 0.134428 | 141 | 0.0047 | 0.2437 |
| KEGG | HC | Biosynthesis.of.siderophore.group.nonribosomal.peptides | NSI | -0.915633 | 0.307754 | 140 | 0.0035 | 0.2437 |
| KEGG | HC | Butanoate.metabolism | NSI | -0.347183 | 0.119422 | 141 | 0.0043 | 0.2437 |
| KEGG | HC | C5.Branched.dibasic.acid.metabolism | NSI | -0.356961 | 0.121822 | 141 | 0.0040 | 0.2437 |
| KEGG | HC | Cell.cycle | NSI | 1.137986 | 0.329979 | 25 | 0.0008 | 0.2437 |
| KEGG | HC | Circadian.rhythm | NSI | 0.570400 | 0.183606 | 16 | 0.0023 | 0.2437 |
| KEGG | HC | Complement.and.coagulation.cascades | NSI | 0.992583 | 0.339503 | 18 | 0.0041 | 0.2437 |
| KEGG | HC | Cytochrome | NSI | 0.884857 | 0.279723 | 16 | 0.0019 | 0.2437 |
| KEGG | HC | D.Alanine.metabolism | NSI | -0.408887 | 0.137868 | 141 | 0.0036 | 0.2437 |
| KEGG | HC | Dilated.cardiomyopathy | NSI | 0.561936 | 0.197398 | 28 | 0.0051 | 0.2437 |
| KEGG | HC | EGFR.tyrosine.kinase.inhibitor.resistance | NSI | 0.852993 | 0.300408 | 21 | 0.0052 | 0.2437 |
| KEGG | HC | Fatty.acid.degradation. | NSI | -0.386585 | 0.135643 | 141 | 0.0051 | 0.2437 |
| KEGG | HC | Gap.junction..PATH | NSI | 0.677898 | 0.229461 | 16 | 0.0037 | 0.2437 |
| KEGG | HC | Global.maps.only | NSI | -0.658729 | 0.224755 | 141 | 0.0040 | 0.2437 |
| KEGG | HC | Glycerolipid.metabolism. | NSI | -0.358175 | 0.122759 | 141 | 0.0041 | 0.2437 |
| KEGG | HC | Hedgehog.signaling.pathway...fly | NSI | 0.519394 | 0.181196 | 17 | 0.0048 | 0.2437 |
| KEGG | HC | Hepatitis.B. | NSI | 1.063585 | 0.322599 | 17 | 0.0013 | 0.2437 |
| KEGG | HC | Hepatitis.C. | NSI | 0.963121 | 0.324891 | 17 | 0.0036 | 0.2437 |
| KEGG | HC | Herpes.simplex.virus.1.infection | NSI | 1.183698 | 0.403583 | 27 | 0.0040 | 0.2437 |
| KEGG | HC | Hypertrophic.cardiomyopathy | NSI | 0.642583 | 0.205224 | 29 | 0.0021 | 0.2437 |
| KEGG | HC | Kaposi.sarcoma.associated.herpesvirus.infection | NSI | 1.216901 | 0.363102 | 41 | 0.0010 | 0.2437 |
| KEGG | HC | Malaria | NSI | 1.060581 | 0.353414 | 21 | 0.0032 | 0.2437 |
| KEGG | HC | Measles | NSI | 1.022219 | 0.318713 | 17 | 0.0017 | 0.2437 |
| KEGG | HC | Mitophagy | NSI | 0.667766 | 0.230826 | 18 | 0.0045 | 0.2437 |
| KEGG | HC | Nucleotide.metabolism | NSI | -0.722554 | 0.241187 | 141 | 0.0033 | 0.2437 |
| KEGG | HC | Peptidoglycan.biosynthesis | NSI | -0.357579 | 0.126263 | 141 | 0.0053 | 0.2437 |
| KEGG | HC | Phosphonate.and.phosphinate.metabolism | NSI | -0.463538 | 0.162135 | 141 | 0.0049 | 0.2437 |
| KEGG | HC | Prenyltransferases | NSI | -0.407991 | 0.136308 | 141 | 0.0033 | 0.2437 |
| KEGG | HC | Propanoate.metabolism | NSI | -0.344081 | 0.115846 | 141 | 0.0035 | 0.2437 |
| KEGG | HC | Protein.processing | NSI | -0.395018 | 0.135438 | 141 | 0.0042 | 0.2437 |
| KEGG | HC | SNARE.interactions.in.vesicular.transport | NSI | 0.786990 | 0.245073 | 18 | 0.0017 | 0.2437 |
| KEGG | HC | Sphingolipid.signaling.pathway | SI | 0.952050 | 0.329819 | 64 | 0.0045 | 0.2437 |
| KEGG | HC | Sulfur.relay.system | NSI | -0.377156 | 0.133305 | 141 | 0.0054 | 0.2437 |
| KEGG | HC | Systemic.lupus.erythematosus | NSI | 0.962602 | 0.312007 | 30 | 0.0025 | 0.2437 |
| KEGG | HC | TGF.beta.signaling.pathway | NSI | 0.793241 | 0.269980 | 19 | 0.0039 | 0.2437 |
| KEGG | HC | Terpenoid.backbone.biosynthesis | NSI | -0.342695 | 0.120078 | 141 | 0.0050 | 0.2437 |
| KEGG | HC | Tight.junction | NSI | 1.070786 | 0.341911 | 37 | 0.0021 | 0.2437 |
| KEGG | HC | Ubiquitin.mediated.proteolysis | NSI | 1.023882 | 0.348257 | 40 | 0.0039 | 0.2437 |
| KEGG | HC | Viral.protein.interaction.with.cytokine.and.cytokine.receptor | NSI | 0.836517 | 0.288481 | 22 | 0.0044 | 0.2437 |
| KEGG | HC | Wnt.signaling.pathway | NSI | 0.731802 | 0.244032 | 22 | 0.0032 | 0.2437 |
| KEGG | HC | mRNA.surveillance.pathway | NSI | 1.025932 | 0.306796 | 17 | 0.0011 | 0.2437 |
| KEGG | HC | Transcriptional.misregulation.in.cancer | NSI | 0.775364 | 0.275270 | 19 | 0.0056 | 0.2483 |

**Notes:**

Differential functional abundance analysis was performed using the R package MaAsLin2 (version 1.15.1). Input abundances were preprocessed by removing features with < 10% prevalence, followed by centered log-ratio (CLR) transformation. Age, sex, BMI, and years of education were adjusted as fixed-effect covariates in the linear model. Multiple testing correction was performed using the Benjamini–Hochberg (BH) method.

The statistical significance threshold for this study was defined as *q* < 0.1. To provide a comprehensive overview of potential functional shifts, a relaxed reporting threshold of *q* < 0.25 was applied for data presentation in this supplementary table. No functional features at the KEGG Orthology (KO), KEGG pathway, or CAZy levels reached the strict significance threshold (*q* < 0.1) across any of the group comparisons (using either HC or NSI as the reference group); features listed herein represent descriptive trends meeting the *q* < 0.25 criteria.

Abbreviations: KO, KEGG Orthology; CAZy, Carbohydrate-Active EnZymes; HC, Healthy Control; SI, Suicidal Ideation; NSI, Non-suicidal Ideation; coef, model coefficient (effect size); stderr, standard error of the coefficient; *p*, nominal *p*-value; *q*, Benjamini–Hochberg corrected *p*-value.
