# Supplementary material for: Novel insights into gut microbiota alterations in major depressive disorder with suicidal ideation: a metagenomic analysis
Source: Front Microbiol. 2026 Jun 10;17:1843301. doi: 10.3389/fmicb.2026.1843301 (PMC13290911; doi:10.3389/fmicb.2026.1843301)
Supplement: Supplementary file 1 [file Supplementary_file_1.zip › Supplementary Table 4.DOCX]

**Supplementary Table 4**. Differential MetaCyc pathways between NSI and SI groups identified by MaAsLin2 analysis.

| Metacyc Pathway | Value | Coefficient | Std. Error | *p*-value | *q*-value (FDR) |
| --- | --- | --- | --- | --- | --- |
| PWY-5265 | SI | −2.91×10^−5^ | 7.78×10^−6^ | 3.40×10^−4^ | 0.0917 |

Note: MaAsLin2 model was adjusted for covariates including age, sex, BMI, and education level. Non-suicidal Ideation (NSI) was set as the reference group. *q*-values were calculated using the Benjamini-Hochberg (BH) method to control the False Discovery Rate (FDR). Abbreviation: SI, Suicidal Ideation; NSI, Non-suicidal Ideation.
